# Metabolomics—A Tool to Find Metabolism of Endocrine Cancer

**DOI:** 10.3390/metabo12111154

**Published:** 2022-11-21

**Authors:** Raziyeh Abooshahab, Hamidreza Ardalani, Maryam Zarkesh, Koroush Hooshmand, Ali Bakhshi, Crispin R. Dass, Mehdi Hedayati

**Affiliations:** 1 Cellular and Molecular Endocrine Research Center, Research Institute for Endocrine Sciences, Shahid Beheshti University of Medical Sciences, Tehran P.O. Box 19395-4763, Iran; 2 Curtin Medical School, Curtin University, Bentley 6102, Australia; 3 Department of Pharmaceutical Sciences, College of Pharmacy, University of Michigan, Ann Arbor, MI 48109, USA; 4 System Medicine, Steno Diabetes Center Copenhagen, 2730 Herlev, Denmark; 5 Department of Clinical Biochemistry, School of Medicine, Shahid Sadoughi University of Medical Sciences and Health Services, Yazd P.O. Box 8915173160, Iran; 6 Curtin Health Innovation Research Institute, Curtin University, Bentley 6102, Australia

**Keywords:** biomarkers, endocrine cancers, early diagnosis, metabolomics, metabolic pathways

## Abstract

Clinical endocrinology entails an understanding of the mechanisms involved in the regulation of tumors that occur in the endocrine system. The exact cause of endocrine cancers remains an enigma, especially when discriminating malignant lesions from benign ones and early diagnosis. In the past few years, the concepts of personalized medicine and metabolomics have gained great popularity in cancer research. In this systematic review, we discussed the clinical metabolomics studies in the diagnosis of endocrine cancers within the last 12 years. Cancer metabolomic studies were largely conducted using nuclear magnetic resonance (NMR) and mass spectrometry (MS) combined with separation techniques such as gas chromatography (GC) and liquid chromatography (LC). Our findings revealed that the majority of the metabolomics studies were conducted on tissue, serum/plasma, and urine samples. Studies most frequently emphasized thyroid cancer, adrenal cancer, and pituitary cancer. Altogether, analytical hyphenated techniques and chemometrics are promising tools in unveiling biomarkers in endocrine cancer and its metabolism disorders.

## 1. Introduction

In spite of decades of research, cancer still ranks among the most common causes of death worldwide, causing not only a huge burden on patients but also on their families and society on a global scale [1]. Oncology experts consider endocrine cancers to be relatively rare and heterogenous tumors with an extensive range of clinical characteristics. Cancers such as thyroid, adrenal, pancreatic, parathyroid, and pituitary are among these types [2]. Endocrine tumors are often misdiagnosed as benign tumors due to their propensity to synthesize hormones, making treatment more intricate [3]. Moreover, in decades of research on endocrine tumors, researchers have discovered that abnormality in hormone production and release can alter the metabolism to develop malignancy. Inhibiting the apoptosis of transformed cells and accelerating proliferation are two possible effects of hormones released by endocrine glands on cancer risk and progression [4]. By way of example, attested as the main regulator of the thyroid gland, thyroid-stimulating hormone (TSH) can promote thyroid tumorigenesis and progression [5,6]. The pituitary gland is responsible for releasing TSH, stimulating the thyroid gland to produce and release hormones called thyroxine (T4) and triiodothyronine (T3), which have key roles in regulating the body’s temperature, metabolism, and heart rate. Other examples include pituitary tumors that produce high levels of adrenocorticotropic hormone (ACTH), which has a correlation with tumor metastasis [7]. By increasing ACTH levels, the adrenal glands produce more cortisol. Apart from that, adrenal tumors release excessive amounts of cortisol that can cause abnormal blood pressure, low potassium levels, and muscle weakness [8]. ACTH can also be produced by other tumors in very rare cases that are associated with similar symptoms [9]. Thus, early diagnosis may improve the chances for successful treatment in this challenging area. In this context, discovering biomarkers associated with metabolic changes and screening methods that differentiate benign from malignant tumors can facilitate practitioners’ selection of effective treatments in an earlier stage.

The omics sciences, including transcriptomics, proteomics, and metabolomics, are developing in the field of oncology and play a critical role in detecting the distinctive patterns of cancer metabolism and identifying specific biomarkers for diagnostic and prognostic of cancer, which ultimately lead to improving therapeutic strategies [10]. An overarching omics science is metabolomics, which is a combination of state-of-the-art-techniques with chemometric analysis tools to provide novel insights into changes in the metabolic pathways involved in diseases present in biological samples [11]. Considering that metabolic reprogramming is one of the hallmarks of cancer cells, metabolomics can be useful for uncovering the link between metabolic regulation and tumorigenesis and subsequently lead to the early detection and diagnosis of cancer, as well as evaluating therapies [11,12].

Metabolites encompass a broad range of chemical properties, having a wide molecular diversity, each belonging to a different class of compounds that interact with phenotypes and reflect the functions of cells and organisms [13]. Their concentrations are affected by a variety of factors such as diet, medicines, exercise, gut microbiome, health condition, hormone levels, gender, and age [11,13,14]. The most common applied biological samples in human metabolomic studies are urine, blood, tissue, and cerebrospinal fluid (CSF), which have a high potential for discovering cancer biomarkers. In metabolic profiling, two major approaches prevail: nuclear magnetic resonance spectroscopy (NMR) and mass spectrometry (MS), coupled with gas chromatography (GC) or liquid chromatography (LC) [15]. Metabolomics experiments can be classified as targeted or untargeted analyses. In targeted metabolomics, it is common practice to conduct a quantitative analysis of a limited set of metabolites, while untargeted analyses attempt to simultaneously determine a large number of metabolites from each sample [13].

It has taken decades for numerous studies to examine the metabolic changes in human samples to understand the pathophysiological mechanisms underlying endocrine cancer development. Considering this, we conducted a systematic search of clinical metabolomics studies on endocrine cancers, focusing on the possibility of using metabolomics to detect changes in endocrine-related tumors and identify metabolic biomarkers and associated pathways.

## 2. Search Strategy and Study Inclusion Criteria

**Figure 1:**
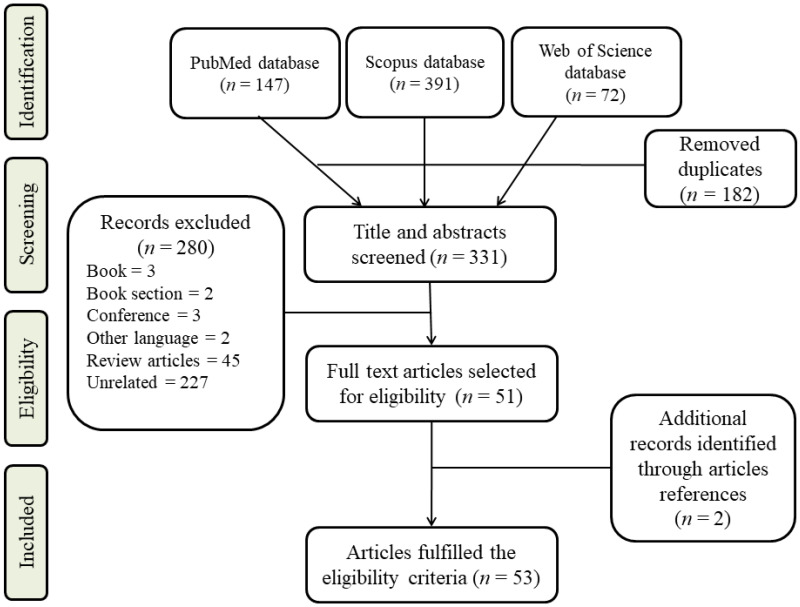
Flow diagram illustrating the process of identifying, screening, and selecting relevant studies.

To conduct this review, PubMed, Web of Science, and Scopus (as online databases) were used to find the most recent articles from 2010 to 2022 related to the subject under investigation. As part of the search strategy, we used the following keywords: ((“metabolomic * AND “Endocrine” AND “cancer” OR “carcinoma” OR “neoplasm”)), ((“metabolomic * AND “Parathyroid” AND “cancer” OR “carcinoma” OR “neoplasm”)), ((“metabolomic * AND “Pituitary” AND “cancer” OR “carcinoma” OR “neoplasm”)), ((“metabolomic * AND (“Adrenal” AND (“cancer” OR “carcinoma” OR “neoplasm”)), ((“metabolomic * AND “thyroid” AND “cancer” OR “carcinoma” OR “neoplasm”))”)) searching for “title/abstract”. Authors carefully examined articles to reduce the bias in selection by checking titles, abstracts, and availability of the articles’ full text. Only English articles having full text were considered. Articles were excluded if they were metabolomics on cell lines, therapeutic strategies, meta-analyses, case reports, or reviews (Figure 1).

**Figure 2:**
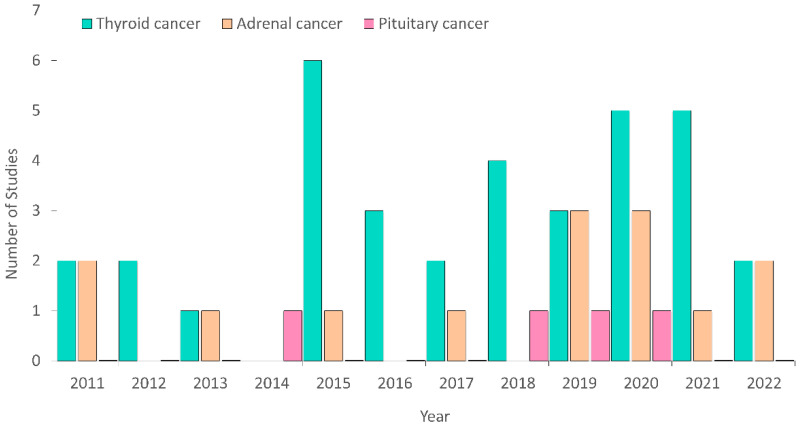
The number of metabolomics studies performed to study various endocrine cancers over the past 12 years.

In our search, thirty-five articles related to thyroid cancer, fourteen articles regarded adrenal cancer, and four articles related to pituitary cancer were found (Figure 2).

### 2.1. Thyroid Cancer

The most common malignancy of the endocrine system is thyroid cancer (TC), ranked seventh among cancers in women [16]. In 2020, there were approximately 586,000 thyroid cancer cases worldwide, with predictions of it reaching 761,000 by 2040, according to the Global Cancer Observatory (GCO) [17]. The histological classification of thyroid nodules comprises four major categories, including PTC, follicular thyroid cancer (FTC), medullary thyroid cancer (MTC), and ATC [18]. Individuals with a history of radiation exposure or residing in iodine-deprived regions and elderly people are more likely to develop thyroid nodules [19]. A fine-needle aspiration biopsy (FNAB) guided by ultrasound with subsequent cytological evaluation is routinely accepted as a diagnostic method for thyroid nodules [20]. However, approximately 15–30% of all FNABs fail to differentiate malignant from benign tumors due to low diagnostic accuracy and producing inconclusive results. Bethesda’s classification classifies this result as atypical or follicular lesions of undetermined significance (AUS/FLUS) (III), and therefore, repeating FNAB is recommended [20,21]. A lobectomy or thyroidectomy may be necessary for diagnosis or treatment in certain cases. Research is currently being conducted to identify early diagnostic biomarkers in order to prevent the need for repeated FNAB and unnecessary surgical operations [22]. By integrating metabolic profiling techniques, it is possible to gain insight into authentically diagnostic metabolites related to thyroid cancer, assist in describing pathological conditions, and improve treatment options.

**Figure 3:**
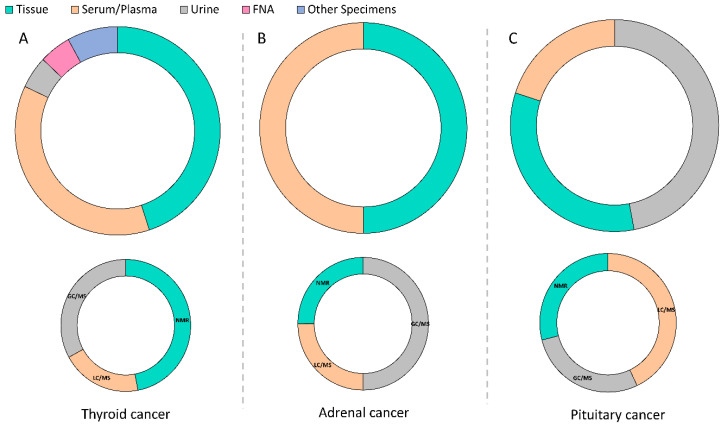
**An overview of endocrine cancers metabolomics studies from 2010 to 2022.** (**A**–**C**): the top row displays the percentage of each sample type used in the reviewed studies, and bottom row represent the percentage of analytical methods used for each endocrine cancer type. Keys: FNA, fine needle aspiration; NMR, nuclear magnetic resonance; GC/MS, gas chromatography-mass spectrometry; LC/MS, liquid chromatography-mass spectrometry.

According to our database search, thirty-five articles from 2010 to 2022 have been published regarding metabolomics in thyroid cancers (Figure 3A). Researchers have primarily focused on using proton nuclear magnetic resonance (1H-NMR)-based metabolomics (17 articles out of 35) with untargeted strategies to discriminate benign thyroid tumors from malignant ones within the past 12 years [23,24,25,26,27,28,29,30,31,32,33,34,35,36,37,38]. A comparison of 1H-NMR results between malignant and benign tumors reveals the following changes in metabolite levels: increased lactate content and amino acids, including glutamic acid, valine, and isoleucine, and decreased choline, myoinositol, scyllo-inositol, and lipid metabolism [24,25,28]. However, a few studies reported contradictory results. Compared to benign samples, malignant lesions contained higher levels of choline, O-phosphocholine in a study by Ryoo et al. who performed NMR analysis of percutaneous FNAB samples aiming to discriminate benign and malignant thyroid tumors, including 35 PTC and 69 follicular adenomas (FA) samples [30]. Furthermore, Zhou and co-workers investigated that the choline content was elevated in patients with PTC [38].

A surge in the use of GC/MS- and LC/MS-based metabolomics has occurred in the past decade to identify metabolic perturbations in thyroid cancers [39]. Eight articles out of thirty-five used GC/MS-based metabolomic approach to discriminate benign thyroid tumors from malignant ones within the past 12 years [40,41,42,43,44,45,46,47]. Moreover, 12 articles out of 35 performed LC/MS-based metabolomics in this area [47,48,49,50,51,52,53,54,55,56,57,58]. In the experiments using those procedures, malignant and benign samples showed different patterns of metabolic changes. As part of a study conducted by Chen et al. metabolomics alterations were analysed through a GC/MS platform in matched PTC tissues and normal thyroid tissues [40]. It was found that metabolites involved in carbohydrate metabolism were downregulated, whereas the metabolism of lipid-based metabolites and nucleotides was upregulated in PTC. Contrary, Wojakowska et al. employed GC/MS to compare various types of thyroid cancers, including FTC, PTC/classical variant, PTC/follicular variant, MTC, ATC, FA, and normal thyroid tissue samples [42]. Malignant samples showed a significant decrement level of metabolites related to long-chain fatty acids metabolism and galactose metabolism compared to control samples and increased lactic acid content. Moreover, PTCs differ from FTC samples by their lower gluconic acid levels and higher citric acid. The presence of increased levels of the decanoic acid ester can further distinguish follicular carcinoma from follicular variants of PTC. As less invasive sample types, Shen et al. compared serum samples from 37 patients suffering from distant metastasis with samples from 40 patients who had undergone ablation using GC/time of flight (TOF)-MS [44]. Metastatic groups showed enhanced alanine, aspartate, glutamate metabolism, and inositol phosphate metabolism. A combination of GC/TOF-MS and LC/quadrupole/time-of-flight (-Q-TOF) approaches was performed by Xu et al. to discriminate between PTC and benign thyroid adenoma (BTA) [47]. The researchers examined 57 cases of PTC and 48 cases of BTA and compared them with the normal group. Both diseases exhibited increased glycolysis and metabolism of amino acids, one-carbon, and tryptophan. Compared with the PTC specimens, taurine, hypotaurine, and purine levels were significantly elevated, while fatty acid and bile acid levels were higher in BTA tissues. Furthermore, Shang et al. combined untargeted and targeted approaches using GC/TOF-MS and ultra-high-performance liquid chromatography coupled with triple quadrupole mass spectrometry (UHPLC/QqQ/MS), respectively to identify the tissue metabolic profile of PTC [43]. A significant finding of their study was galactose metabolism pathways that could directly influence PTC development by modulating energy metabolism, which was significant in both GC/MS and LC/MS analyses. In two individual studies conducted by our group, plasma metabolic perturbations of PTC, multinodular goiter (MNG), and MTC, as well as a healthy group, were evaluated. The results indicated that linoleic acid, phenylalanine, arachidonic acid, glycine, D-glutamine, D-glutamate, and GSH, have common influences on both PTC and MNG tumorigenesis [45]. Metabolism alterations in MTC were predominantly associated with the biosynthesis of unsaturated fatty acid and amino acid metabolism, mostly glutamine and glutamate metabolism [46]. Yao et al. evaluated 140 serum samples from PTC (*n* = 30), NG (*n*  =  80), and healthy controls (*n*  =  30), using LC coupled with linear ion trap quadrupole (LTQ) orbitrap mass spectrometry [48]. Between benign and malignant nodules, the major alteration in metabolites occurred in lipid metabolism, primarily 3-hydroxybutyric acid. In one of those studies, Guo et al. used exhaled breath as a non-invasive sample type, collected from 64 patients, to investigate the metabolism of thyroid cancer by analysing the volatile organic compounds (VOCs) [41]. In the healthy and MNG groups, metabolite levels of sulfurous acid, cyclohexylmethylhexylester, isolongifolene-5-ol, 3,5-decadien-7-yne, and cyclohexanone had significant differences. Additionally, PTC responded statistically differently from healthy individuals in the presence of several key metabolites, such as cyclohexanone, 4-hydroxybutyric acid, phenol, 2,2-dimethyldecane, and ethylhexanol.

NMR spectroscopy research has become more apparent in TCs, with the results pointing to metabolic insights into the disease. Meanwhile, LC/MS- and GC/MS-based metabolomics enable simultaneous analysis of many polar, semi-polar and nonpolar metabolites, resulting in greater metabolic transparency. Lactate, choline, mono- and disaccharides, and TCA intermediates in TCs are well documented [24,25,30,45,58]. The contradictory results among the published papers regarding TCs can be attributed to different study designs, the use of different instruments and/or chemicals, different extraction methods, the confounding effect of race, genetics, age, and the other factors that influence metabolite levels. However, it cannot be denied that a wider information set for metabolic markers needs to be collected, and further work needs to be done on TC metabolomics.

### 2.2. Adrenal Cancer

The adrenal gland is an integral part of the endocrine system, located in the upper part of the abdomen with the outer and inner parts, which produce steroid hormones and catecholamines, respectively [59], leading to the regulation of carbohydrates, protein, and fat metabolism. Adrenal cancer is categorized into three groups: adrenal cortex tumors, the adrenal medulla, and extra-adrenal paraganglia tumors [59,60]. It is estimated that 2% of humans develop adrenocortical carcinomas (ACCs), and 50% of these people survive beyond five years [61]. Several molecular changes are thought to be responsible for ACCs, including tumor suppressor gene inactivation, oncogene activation, DNA mutations, and epigenetic changes [62]. Oftentimes, it is difficult to distinguish between atypical adrenal cortical adenomas (Ads) and ACCs when neither is overtly malignant [61]. The Weiss scoring system recognizes three or more morphological parameters as indicators for malignancy [63]. Nevertheless, it is not precise in borderline cases where only one or two Weiss criteria are met; consequently, malignancy cannot be confirmed [64]. Hence, the search for promising measuring methods to increase the accuracy of malignancy diagnosis in these types of endocrine cancers is of great interest.

According to our database search, fourteen articles from 2010 to 2022 were published regarding metabolomics in adrenal cancers (Figure 3B). Over the past 12 years, researchers have primarily focused on distinguishing ACC from adrenocortical adenoma (ACA) in urine steroids using mass spectrometry-metabolomics [65,66,67,68,69,70,71], and fewer studies were performed on plasma/serum or tissue [64,72,73,74,75,76,77].

An analysis of 24-h urine samples from ACA and ACC patients, along with healthy controls (26 men, 62 women) by GC/MS in selected-ion-monitoring mode was done by Arlt et al. which their findings indicated tetrahydro-11-doxycortisol (THS) as the most highly discriminating marker between ACC and ACA [65]. Patel et al. used unbiased ultra-performance liquid chromatography/mass spectrometry (UPLC/MS) to analyse fasting urine samples from 19 patients with ACC and 46 patients with benign adrenal tumors [67]. They discovered 69 features, which among them creatine riboside levels in the urine were found to be 2.1-fold higher in patients with ACC. Meanwhile, L-tryptophan, N-trimethylysine, and 3-methylhistidine levels were lower in patients with ACC. Velikanova et al. reported GC/MS-based metabolomic analysis performed on urine steroids obtained from 25 patients with Cushing’s syndrome, in which 12 patients with adrenal neoplasms had malign potential (Weiss 1–3), and 24 patients with adrenocortical adenoma considered as a control group [69]. Based on their findings, they suggest that steroid metabolism changes can serve as early indicators of malignancy within the context of Cushing’s syndrome.

Using another biological specimen, researchers examined the metabolic profile of 66 tissue samples from patients diagnosed with Ad, ACC, and pheochromocytoma (PCC) using the ^1^H-high-resolution magic-angle spinning nuclear magnetic resonance (HRMAS NMR) spectroscopy technique. A significant difference was observed between ACCs and Ads in the levels of lactate, acetate, and total choline-containing compounds measured in their study [64].

Schweitzer et al. recently examined the utility of liquid chromatography-tandem mass spectrometry (LC/MS-MS) profiling of plasma steroid hormones in diagnosing adrenocortical tumor malignancies. The steroid hormones of 66 ACA and 42 ACC were quantified in plasma samples [73]. Progesterone, 17-hydroxyprogesterone, 11-deoxycortisol, dehydroepiandrosterone (DHEA), dehydroepiandrosterone sulfate (DHEAS), and estradiol were found to be significantly higher in ACC than in ACA.

The term ‘adrenal incidentalomas’ (AIs) refers to other types of adrenal tumors, found in approximately 4 to 10% of individuals over the age of 50 during routine screenings [60]. The clinical method used to assess incidentalomas is often insufficiently sensitive and unable to distinguish between a hormone-functioning, malignant mass, and a benign mass. A group of scientists examined 19 major steroids and their metabolites in 58 urine samples from patients with non-functioning AIs and healthy subjects using both GC/MS and GC equipped with a flame ionization detector (FID) [66]. They concluded that cortisol, tetrahydrocorticosterone, tetrahydrocortisol, allotetrahydrocortisol, and etiocholanolone can be used as biomarkers to detect AIs.

Pheochromocytomas and paragangliomas (PGLs) are other kinds of adrenal tumors that also belong to rare neuroendocrine tumors that originate in the adrenal medulla and sympathetic or parasympathetic nerves [60]. Germline mutations are estimated to influence 40 percent of these tumors, whether hereditary or somatic. On the basis of their expression profiles, PPGLs can also be grouped into two main clusters as follows: cluster 1 exhibits pseudohypoxia and contains mutations in von Hippel-Lindau (VHL), succinate dehydrogenase (SDHx) and endothelial PAS domain protein 1 (EPAS1), cluster 2, is recognised by the tyrosine kinase receptor activation and mutations in RET and neurofibromatosis type 1 (NF1) [78]. Tumor catecholamine content, secretory rate, and catecholamine biochemical phenotype can all be used to separate clusters (adrenergic or nonadrenergic) [79].

In a study by Rao et al., homogenates of 32 sporadic PGLs and 48 PGLs from patients with mutations in *SDHB*, *SDHD*, *SDHAF-2*, *VHL*, *RET*, and *NF-1* were underwent 1H-NMR analysis for untargeted and LC/MS-MS for targeted metabolite profiling [72]. Sporadic tumors commonly contained lactic acid, acetic acid, and catecholamines. Succinic acid concentration was high in SDHB tumors (9.89 nmol/mg tissue). RET tumor exhibited high epinephrine resonance and overlap between three doublets derived from ATP/ADP/AMP. In a study published by Martins et al. metabolic profile of 24-h urine samples of *SDHx* mutation carriers with tumors, (affected mutation carriers), without tumors (asymptomatic mutation carriers), and patients with sporadic pheochromocytomas and PGLs using NMR analysis were compared [68]. There were considerably differences in the metabolite composition in patients with SDHx-associated neoplasia and sporadic PPGL patients in terms of gluconeogenesis, pyruvate metabolism, ammonia recycling, porphyrin, and aspartate metabolism. Bliziotis et al. conducted a study by performing NMR-based untargeted metabolomics analysis on plasma samples from 36 PGL patients [77]. In their results, tyrosine levels were significantly different among patients with high and low body mass index (BMI). After comparing preoperative and postoperative samples, a metabolic signature including ketones, glucose, organic acids, methanol, dimethyl sulfone, and amino acids was found.

It is thus now more apparent that 24-h urine steroid metabolite excretion in patients with adrenal tumors can be used to distinguish patients with indeterminate tumors. Apart from steroid metabolites, there is still room for investigation of other metabolic changes using different matrices.

### 2.3. Pituitary Adenoma

Pituitary adenomas (PAs) are benign tumors that grow abnormally in the anterior pituitary gland [80,81], with usually no clear cause or reason behind pituitary adenomas development. Depending on their size or origin, they can be classified as microadenomas (less than 10 mm), macroadenomas (over 10 mm), or giant tumors (greater than 40 mm) [82]. It has been noted that some operating pituitary adenomas have a cell type that increases the production of hormones from the anterior pituitary [82]. When adenoma fail to function properly, it may compress portions of the pituitary gland, which may result in hormonal deficiencies [80]. In order to diagnose pituitary adenoma, a multidisciplinary team should be assembled comprising an endocrinologist, an ophthalmologist, and a neurosurgeon [80]. The Endocrine Society clinical practice guidelines recommend magnetic resonance imaging (MRI), biopsy, and complete hormonal assessment in blood and urine, even in asymptomatic patients including prolactin, TSH, free T4, follicle-stimulating hormone (FSH), Insulin-like growth factor 1 (IGF-1), growth hormone (GH), ACTH, estradiol, testosterone, bone morphogenetic protein (BMP), and fasting early morning cortisol to confirm the disease [83]. Nonetheless, the diagnosis of PA is challenging, resulting in treatment delays that might negatively affect the outcome of this disease. Consequently, early detection and improved treatment strategies require the identification of biomarkers and understanding of the metabolism of this cancer.

In terms of metabolomics to find metabolic perturbation of PA, our search resulted in the discovery of four articles over the past 12 years [84,85,86,87] (Figure 3C). Among them, GC/MS was used in two study designs, and LC/MS and NMR were each performed in one study.

Oklu et al. examined plasma samples from patients undergoing ipsilateral (IPS) (*n* = 7) compared them to contralateral samples and two samples from those who were not found to have ACTH hypersecretion (*n* = 9) using LC/MS-MS [84]. They reported 12 metabolites were significantly changed in patients with ACTH-secreting PA compared to the control group; however, after *p* value correction, only 3 metabolites remained significant including deoxycholic and 4-pyridoxic acids and 3-methyladipate. Feng et al. conducted a metabolomics and proteomics study in a group of patients with ACTH-secreting PA [85]. To analyze metabolomes, brain tissue samples from 6 patients with ACTH-secreting PA were compared with 7 subjects with normal pituitary gland. As a result, short-chain fatty acids including heptanoic acid, octanoic acid, nonanoic acid, hexanoic acid were upregulated, and glucose-6-phosphate was downregulated. In another study on 56 tissue samples from patients with confirmed pituitary adenomas and seven normal pituitary glands [86], all pituitary adenomas displayed impaired glucose metabolism and glycolysis when compared to normal tissues.

More recently, Ljare et al. performed 1H-NMR based metabolomic analysis of serum and whole-blood among three groups: luteinizing/follicle-stimulating (LH/FSH)-secreting (*n*  =  24), prolactinomas (*n*  =  14), and non-functional (NF) (*n*  =  9) tumors [87]. In comparison to the LH/FSH-secreting tumor group, prolactinomas showed elevation in beta-hydroxybutyrate (BHB) level only in the serum. Additionally, phenylalanine levels in NF tumors were higher in serum compared to prolactinomas. NF tumors displayed elevated levels of alanine, tyrosine, and formate, but none of these levels demonstrated a statistically significant difference from prolactinomas.

Whilst these limited studies did not confirm the metabolic alterations of pituitary adenoma, it did partially substantiate the perturbation in metabolic pathways including biosynthesis of free fatty acids and glycolysis.

## 3. Conclusions

**Figure 4:**
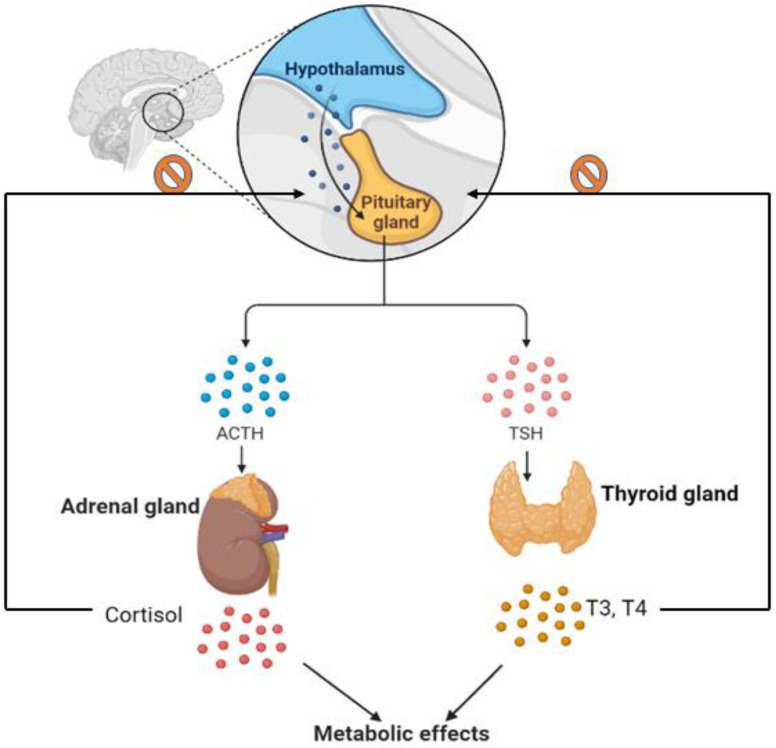
**Relation of endocrine glands**. As part of its function, the pituitary gland releases thyroid stimulating hormones (TSH) and adrenocorticotropin hormones (ACTH). By doing so, TSH and ACTH trigger thyroid and adrenal glands to produce and release hormones. The level of thyroid and adrenal hormones in the blood can be increased, thereby increasing metabolic rate. ACTH and TSH are no longer produced by the pituitary when thyroid and adrenal hormones reach a threshold. Negative feedback ensures that thyroid and adrenal hormones remain within normal ranges.

Although metabolomics is still under development, there are indications that it could be an invaluable tool for the early detection of endocrine cancers by monitoring fluctuations in metabolite concentrations. In this area, several metabolic candidates have been introduced as potential biomarkers for endocrine cancers by using high-throughput techniques. However, there are no comprehensive and adequate metabolomics studies addressing the fluctuations in metabolites in endocrine cancers and none of them adopted in clinics, due in part to the challenge of understanding the aetiology of these disorders. In essence, the changes in metabolites levels may be attributed to the level of consumption or to a change in their regulation based on genetics, diet, lifestyle, environment, stress, age, amongst other factors. Moreover, it is evident that the endocrine glands interact and influence the metabolism of the body by releasing hormones and controls each other during negative and positive feedback (Figure 4). To overcome these challenges, screening other glands and the levels of the hormones before metabolomics study regarding each endocrine cancer, conducting in-depth comprehensive metabolome analysis using different matrices, and decreasing the effect of confounding factors may allow a deeper understanding of the relationship between the systemic metabolic abnormalities and endocrine tumorigenesis. In general, understanding molecular cancer mechanisms will be aided by metabolomics, which could be a unique strategy of using universal approaches in the early diagnosis of endocrine cancers, distinguishing between benign and malignant lesions, and developing precision and targeted therapies.

## Data Availability

All raw data is available by sending a request to the corresponding author, or R.A.
